# Assessment of prerequisite programs implementation at food packaging manufacturing companies and hygiene status of food packaging in a developing country: Cross-sectional study

**DOI:** 10.1016/j.heliyon.2023.e19824

**Published:** 2023-09-04

**Authors:** Marilyn Abdessater, Fady Fayyad, Joseph Matta, Layal Karam

**Affiliations:** a Faculty of Nursing and Health Sciences, Notre Dame University Zouk Mosbeh, Lebanon, P.O. Box: 72, Zouk Mikael, Lebanon; b Industrial Research Institute, Lebanese University Campus, Hadeth, Baabda, Lebanon; c Department of Nutrition, Faculty of Pharmacy, Saint-Joseph University of Beirut, Medical Sciences Campus, Damascus Road, P.O.B. 11-5076, Riad Solh, Beirut, 1107 2180, Lebanon; d Human Nutrition Department, College of Health Sciences, QU Health, Qatar University, Doha, Qatar

**Keywords:** On-site inspection, Packaging contamination, Acceptance criteria, Contact surface, Hand swab

## Abstract

Food packaging has a critical role in all food types and along the food chain from product preservation to transportation, distribution, storage, retailing, and end-use. However, it can become a source of contamination and transfer of microorganisms to the packed food when its hygienic status is not well maintained. The aim of this study was to evaluate the Prerequisite programs (PRPs) implementation in 5 food packaging companies across Mount Lebanon through on-site inspections and to assess the compliance of contact surfaces, employee hands and packaging materials to microbiological specifications. Following on-site inspection, none of the companies achieved a full total score of 100% and scores ranged from 25 to 62%. Regarding the assessment of hygienic status of contact surfaces, non-conforming results (acceptable limit ≤0.6 log colony forming units (CFU)/cm^2^) were observed in 50% (5/10) of the surfaces for total viable count (TVC). For the employee hands, none of the hand swab samples (10/10) was conforming for TVC that was present in all samples above the acceptable limit. Highest and lowest reported values were 4.4 and 1.7 log CFU/hands respectively. For packaging samples collected during on-site inspections, TVC and yeasts and molds were detected in 20% (2/10) of the samples. However, the samples collected from the retail market, had higher contamination rates of 95% (19/20) and 65% (13/20) for TVC and yeasts and molds, respectively. As for *Enterobacteriaceae,* it was not detected in all tested contact surfaces, employees' hands, and packaging samples. PRPs assessment and related verification activities showed the need for companies to strengthen their hygienic programs and highlighted the importance of food safety management systems (FSMS) implementation not only in food companies but also in food packaging companies. Additionally, the effectiveness of PRPs implementation should be assessed on planned routine basis.

## 1 Introduction

Food packaging is defined as a coordinated system of preparing food for transport, distribution, storage, retailing, and end-use [1]. The main role of packaging is to hold, protect and preserve food product integrity against potential damage from climatic, microbiological and transit hazards [2]. However, the packaging itself can become a source of food contamination when its hygienic status is not well maintained. Previous studies showed that packaging material such as paper and paper boards were contaminated by high bacterial counts and yeasts and molds [[3], [4], [5]]; in addition to contamination of cling film by *Bacillus* species (spp.), *Staphylococcus* aureus, *Klebsiella* spp [6]. During cheese production, samples were collected from the cheese just before packaging, from packaging containers and from cheese after packaging. It was found that the level of contamination in cheese increased for aerobic plate count, aerobic spore-formers, coliforms, *Staphylococci*, *Enterococci,* after packaging in plastic container and cardboard laminated sheets [7]. In addition, inoculated reusable plastic containers and cardboard contaminated packed peaches with *Escherichia coli, Pseudomonas,* and *Saccharomyces cerevisiae* [8]. When contaminated packaging is in direct contact with food, it can transfer spoilage microorganisms and/or pathogens to the product causing quality deterioration, food waste, shorter shelf-life and foodborne illnesses. Potential sources of contamination include: processing environment, type of circulation water, surface contact, working practices (Good Hygiene Practices), pest infestation, storage of raw material and food packaging material [9]. This is the reason why several national and international standards were developed and enforced for food packaging material manufacturing such as British retail consortium.

(BRC) for packaging and packaging material, international organization for standardization (ISO) 22,000:2005 technical specification ISO/Technical Specification (TS) 22,002–4, USA's Food and Drug Authority (FDA) food safety modernization act (FSMA), European Union's (EU) European Commission (EC) No. 1935/2004, and Lebanon's Lebanese standards institution (LIBNOR) Lebanese conformity English (NL EN) 13,427:2007 [10] and NL EN 15593:2012 [11]. Even though these standards are available, they are not a regulatory requirement and there is no baseline information on the extent of adherence to them by Lebanese food packaging companies. Based on those requirements, the hygienic status is assessed by prerequisite programs (PRPs) evaluation and verified by objective measurements. PRPs assessment is based on the evaluation of: establishment layout and workspace, utilities, waste disposal, equipment suitability, cleaning and maintenance, management of purchased materials and services, measures for prevention of contamination, cleaning, pest control, personnel hygiene and facilities, rework, withdrawal procedures, storage and transport, food packaging information and customer communication, food defense and bioterrorism [12]. Hence the need of inspection, audit, routine monitoring and verification methods of PRPs. Objective measurements can include testing of contact surface, employee hand, packaging material, ambient air, and water. Total aerobic count and *Enterobacteriaceae* are common indicators for the three objective measurements in addition to *Staphylococcus aureus* for employee hand testing and yeast and molds for food packaging material [4,5,[13], [14], [15], [16], [17]]. Studies in the literature showed that PRPs evaluation were mainly carried out in catering and food production [13,[18], [19], [20], [21], [22], [23], [24]]. However, despite the importance and impact of packaging on all food sectors, no published studies were available in food packaging companies. The aim of this study was to evaluate the PRPs implementation in food packaging companies across Mount Lebanon and to assess the compliance of contact surfaces, employee hands and packaging materials to microbiological specifications.

## 2 Materials and methods

### 2.1 Food packaging companies

A cross-sectional study was conducted throughout February 2020 till July 2020 targeting food packaging companies across Mount Lebanon. Five companies were chosen as a probability random sample of registered food packaging companies in Mount Lebanon. The characteristics of the companies are shown in Table 1. An initial contact with the top managers of the selected companies was scheduled (mobile conversation followed up with an email). Each company was visited by the same trained person in food safety and PRPs, in order to conduct the face-to-face interviews, carry out the inspection and collect the samples. The duration of the visit was approximately 1–2 h.

**Table 1:** Company characteristics of food packaging companies (n = 5).

Company	Packaging Type	Intended Use	Food Application	Quality/food safety management system
**A**	Plastic Bottles, gallons	Ready to eat (RTE)	Ketchup & Lemon Substitute Juice	ISO 9001:2015
**B**	Bags, flexible packaging	Freezing, vacuum packaging, modified atmosphere packaging, RTE	Bakery products, cereals & spices, nuts, seafood, coffee, dairy food, fresh & frozen food	None
**C**	Bags, flexible packaging, wrappers, pallets	Heating, freezing, microwave, boil in bags, vacuum packaging, modified atmosphere packaging, collation shrinking, RTE	Bakery products, candy & chocolates, cereals & spices, dry food, nuts, seafood, coffee, dairy food, chips, fresh & frozen foods, instant drink, pet food packaging	None
**D**	Trays, plastic containers	Heating, freezing, microwave, re-use, RTE	Bakery products, candy & chocolates, spices, dry food, nuts, dairy food, fresh & frozen foods, instant drink	ISO 9001:2015
**E**	Flexible packaging, wrappers	Freezing, vacuum packaging, modified atmosphere packing, RTE	Bakery products, candy & chocolates, cereals & spices, dry food, nuts, seafood, coffee, dairy food, chips, fresh & frozen foods, instant drink	None

Data collection took place after obtaining the ethical approval of the International Review Board (IRB) at Notre Dame University (NDU); approval number (Ref #:IRBSP2019_3_FNHS). A written informed consent form was signed by the companies' staff participating in the study.

### 2.2 Questionnaire

The developed questionnaire consisted of two parts. The first part covered the general company characteristics and was adapted from De Boeck et al. [25]. It described information related to the company production sector, intended use/application, certification(s), etc. The second part of the questionnaire included an inspection checklist prepared according to Libnor standard NL EN 15593:2012 [11] and ISO/TS 22002–4:2013 [12].15 main clauses were thus evaluated and included: establishment layout and workspace, utilities, waste disposal, equipment suitability, cleaning and maintenance, management of purchased materials and services, measures for prevention of contamination, cleaning, pest control, personnel hygiene and facilities, rework, withdrawal procedures, storage and transport, food packaging information and customer communication, food defense and bioterrorism. In order to enable a differentiated assessment, each clause/sub-clause was scored according to the following four situational descriptions of performance levels: No compliance (score 0/2), partial compliance (score 1/2), full compliance (score 2/2) and Not applicable (N/A) (score 0/0). Scoring of each main clause was obtained based on total score of its sub-clauses or its own score in the case of absence of sub clauses. The total score was obtained by adding all the scores of the main clauses using Microsoft Excel. Both main clauses and total scores were converted to a score over 100 and calculated into percentages rounded to the nearest whole number.

### 2.3 Collection of hand and surface swab samples

Hand and surface swab samples were collected according to ISO 18593:2004 [26]. Hand samples from packaging handlers were collected from both hands during regular working activities from two workers directly handling packaging material at the end point of the active production line during the visit and before warehouse storage or distribution [13,17]. Similarly, surface swab samples were collected from surfaces in direct contact with packaging material at the end point of the production line during operation and before storage [13]. Target contact surface areas were delimited by a stainless-steel sterilized template of 25 cm^2^ and swabbed using a sterile cotton wool swab (Citoswab; China) moistened with transport-neutralized solution of Letheen broth (Scharlau; Spain) [26]. Surfaces were swabbed for 20 s and then introduced into the transport tubes.

### 2.4 Collection and preparation of packaging samples

Convenient packaging samples were collected from both the inspected companies and the retail market. A total of ten samples were collected from the 5 visited companies. In each company, two packaging samples were obtained from the warehouse, representing the entire production of food packaging at the time of the visit. Additionally, 20 samples were obtained from a representative retail packaging distribution company that supplies several catering and food companies in different areas of the country. Four packaging samples were selected from each of the following 5 categories: dairy/ice cream containers, salad/bakery/meat containers, ready to eat (RTE) mezze containers, sauce cups with lids (<100 ml) and dessert/fruit cups with lids. The criteria for category selection were risk-based and included packaging intended to be used without further sterilization or cleaning before coming in contact with food or intended for packaging perishable high risk products, or ready-to-eat food with a shelf-life of several days to few weeks [27]. Flexible plastic packaging samples were swabbed from the inside as described for contact surfaces in section 2.3. Rigid Plastic packaging containers were tested using the rinse method. Each container was rinsed with 300 ml of Letheen broth (Scharlau; Spain) then shaken for 30 s. The liquid was then filtered using Cellulose Nitrate (CN) membrane filter 0.45 μm (Sartorios; Germany) and passed through 47 mm filtration assembly (Wheaton, USA) using pressure differential vacuum of 400 mm Hg (Corning; USA). CN was then placed on agar media specific to the target microorganism to be tested (section 2.5).

### 2.5 Microbiological analysis

Collected samples were immediately transported to the laboratory in containers with ice and kept refrigerated (1–4 °C) until the start of the microbiological analysis (<4 h from collection). Tests were carried out at the Industrial Research Institute (I.R.I.) which is accredited by American national standards institute (ANSI) national accreditation board (ANAB) lab for international laboratory accreditation cooperation mutual recognition arrangement (ilac-MRA).

Contact surfaces and hand samples were analyzed for total TVC and *Enterobacteriaceae* while packaging samples were additionally tested for yeasts and molds [28]. The filter from the rinse method or 10-fold dilutions from the transport liquid of the swab samples were plated on appropriate agar media. TVC was enumerated on tryptic soy agar (TSA) (CASO agar; Merck) and incubated for 72 ± 3 h at 30 ± 1 °C. *Enterobacteriaceae* enumeration was on Violet Red Bile Glucose Agar (VRBG) (Scharlau; Spain) after incubation for 24 ± 2 h at 37 ± 1 °C and for yeasts and molds, Sabouraud Dextrose Agar (SDA) (Bio-Rad,USA) was used for 5 days at 25 °C. Tests were conducted in replicate and results were reported as mean values ± standard deviation (SD). All colonies were counted on each plate and counts per sample were reported as mean CFU per cm^2^ for contact surface and flexible packaging, per employee hands swabs and per container for rigid plastic packaging. Values were then converted to logarithms of the number of CFU per respective unit (cm^2^, hands, container).

## 3 Results and discussion

### 3.1 Overall compliance to PRPs requirements

Following on-site inspection, none of the companies achieved a full total score of 100% according to the requirements of the ISO 22002–4: 2013 standard [12] related to prerequisite programs for food packaging manufacturing (Table 2). Total scores for companies ranged from the highest being 62% (company D) to the lowest being 25% (company A) (Table 2). Remaining scores for companies C, B and E were 56%, 53%, and 51% respectively (Table 2). It is noteworthy that the companies with the highest and lowest scores had a quality management system (QMS) certification for ISO 9001, while the 3 other companies didn't have any quality or safety system (Table 1). However, those scores cannot be correlated to the presence or absence of QMS, since ISO 9001 does not address PRPs related to ISO/Technical Specification (TS) 22,002–4. The QMS in this case has no impact on GMP scores. Similar results were seen for catering services located in northern Spain (n = 15) where none achieved the maximum score (43), with total scores ranging from 19 to 36 points [13]. In addition, a study conducted on government and private hospitals evaluation in Ankara, Turkey (n = 20) also had similar results where the overall mean score on the prerequisite questionnaire for hospital food service was 57.5% [18].

**Table 2:** PRPs checklist compliance scores.

Company Compliance Scores (%)					
Clause	A	B	C	D	E
4.1 Establishment	60	100	30	50	60
4.2 Layout and workspace	39	58	33	47	47
4.3 Utilities	20	53	27	33	43
4.4 Waste	33	50	42	92	50
4.5 Equipment suitability, cleaning and maintenance	27	42	58	42	50
4.6 Management of purchased materials and services	69	50	69	100	81
4.7 Measures for prevention of contamination	0	39	64	41	64
4.8 Cleaning	17	50	58	50	67
4.9 Pest Control	19	87	94	94	44
4.10 Personnel hygiene and facilities	7	14	45	57	43
4.11 Rework	21	N/A^a^	100	85	N/A^a^
4.12 Withdrawal Procedures	25	75	50	50	50
4.13 Storage and transport	32	59	71	73	73
4.14 Food packaging information and customer communication	33	87	100	100	25
4.15 Food defence and bioterrorism	12	12	0	37	12
Total Score	25	53	56	62	51

a Not applicable (N/A).

Out of the five companies assessed, 20% achieved full compliance (100% score) for clauses related to establishment, management of purchased materials and services, rework and 40% achieved full compliance for clause food packaging information and customer communication (Table 2). Non-compliance (0% score) was registered for 20% of the

Companies for clauses related to measures for prevention of contamination and food defense and bioterrorism (Table 2). Partial compliance was observed in all companies for the remaining clauses and the scores ranged from 7% to 94% (Table 2). Similarly, in food businesses assessed in Turkey (n = 109), PRPs proper food safety practices were often not being followed in many food businesses i.e., personal hygiene, equipment cleaning and sanitation, and general sanitation procedures [19].

Company A is specialized in production of plastic bottles and gallons for food and non-food applications (chemicals and detergents). However, the quality objectives focused mainly on product density and thickness rather than safety or hygienic status of packaging. The lowest score was full non-compliance (0% score) for clause related to measures for prevention of contamination (Clause 4.7, Table 2), and the highest was 69% for management of purchased materials and services (Clause 4.6, Table 2). The facility was an old building located within close vicinity to industrial zone and did not keep all areas within boundaries in conditions that will protect against contamination. Those may be some of the factors that can explain the lowest total score for this establishment (Table 2).

On the other hand, company B, was a recent building in addition to the construction of a newer facility. It showed full compliance (100%) to the clause related to establishment (Clause 4.1, Table 2) by giving full consideration to the potential sources of contamination from the local environment in its new building and was fully protected against sources of contamination. The lowest score was for clause of food defense and bioterrorism 12% (Clause 4.15, Table 2). The company specialized in food packaging production, had a big portion of the market share, had more staff members with specified departments and the production manager was in charge of quality.

Company C was also located within close vicinity to industrial zone and did not keep all areas within boundaries in a condition that will protect against contamination. However, it showed full compliance (100%) to clauses related to rework (storage, identification and traceability, and usage), food packaging information and customer communication (Clauses 4.11 & 4.14, Table 2). Non-compliance (0% score) was recorded for food defense and bioterrorism (Clause 4.15, Table 2). The plant manager had full awareness of the clauses but explained the difficulty of implementing them, due to budgeting reasons. Most matters that did not incur additional costs and required staff effort such as research, documentation was in partial or full compliance.

Company D had the highest score and achieved full compliance (100%) for food packaging information, customer communication and management of purchased materials and services (Clauses 4.6 & 4.14, Table 2). Lowest score was observed for the utilities clause 33% (Clause 4.3, Table 2). The company took part in European tradeshows and had

Certified international suppliers and customers that set specific requirements that were met. Management commitment was noticeable and willingness for future certifications was in the pipeline.

Company E had specialized personnel and the quality manager showed strong knowledge of the standard. In addition to standard requirements, there was deep knowledge by the quality manager of chemical properties of all raw material used, tests that need to be conducted, and safety of solvents used. Entry to the facility was not granted without permission, and facility layout was properly segregated within limited space given. Highest score was achieved for management of purchased materials and services 81% (Clause 4.6, Table 2); and the lowest one was for clause of food defense and bioterrorism 12% (Clause 4.15, Table 2). In addition to system documentation requirements, needed certificates such as treatment certificate for wooden pallets were present only in company E. All necessary documentation was present, and this facilitated data transfer and answering several questions related to standard requirements.

### 3.2 Assessment of compliance to specific PRPs requirements

Regarding layout and workspace companies B and E (40 %) were spacious enough to allow a logical flow of materials, products and people through the production process. Similarly, adequate facilities for meals production were found in all food units evaluated in schools (n = 88) at Portugal [21]. None of the companies had drains, cleaning with water was not part of the routine process and when used it was swept in the direction outside the company towards the nearest entrance/exit. Additionally, standing water was present on-site in companies A and D (40%) (Table 3). Company A (20%) had a dedicated separate floor for storage of wrapped raw material and shrink-wrapped finished products protecting them from dust and contamination with proper segregation of raw materials, intermediate materials, chemicals and finished food packaging (Table 3). Similar compliance was found in all school foodservice evaluated where food storage in adequate containers and freezing and refrigeration temperature documentation were good practices [21]. The remaining companies did not store raw material rolls far enough from production zones and other sources of contamination. Companies B and D (40%) took some preventive measures (pallets, wrapping, removing first side of the roll that was exposed to the environment) while the remaining companies C and E (40%) did not (Table 3). Similar to our study for catering services in northern Spain, the most important non-conformities were the insufficient storage space for foods (60%) and repeated deviations were not enough storage capacity in warehouses, and poor stock rotation (66.7%) [13]. In addition, all great chain restaurants in Iran had poor condition for storage [23], and inadequate number of storerooms were seen in foodservice at hospitals in Turkey [18]. Since the foodservice setting at hospitals is different than the one in food packaging industries, we compared only the main PRPs that are not sector specific and are required for all establishments in the food chain.

**Table 3:** Summary of main non-conformities found in inspected companies (n = 5).

Main non conformities	Company A	Company B	Company C	Company D	Company E
Standing water on-site	P	P	P	P	P
No established requirements for air used in direct contact with food packaging material	P	P	P	P	P
Did not store raw material rolls far enough from production zones and other sources of contamination.	A	P	P	P	P
No clearly identified bins to differentiate between production and non-production waste	P	P	P	P	P
No established requirements for water and separate supply of potable water	P	P (only Installed reverse osmosis water purification device)	P	P (only Installed reverse osmosis water purification device)	A
Non-suitable equipment hygienic design and food packaging contact surfaces	P	P (no measures to control leather and scotch tape on the final counters)	P	P	P (no measures to control wooden surfaces covered with nylon)
No preventive maintenance plans and maintenance performed only when needed	P	P	A	A	A
No implemented measures for measures for prevention of contamination	P	P	P	P	P (carried out and documented a hazard analysis; but was not fully applied)
No cleaning schedule or monitoring program	P	P	A	A	P (inadequate cleaning monitoring program)
Non-compliant policies, documentation, records of used materials for pest control, pest proofing	P	P (incomplete pest proofing)	P (incomplete pest proofing)	P (incomplete pest proofing)	P
No procedure in place for personnel, visitors and contractors	P	A	P	A	A
No hand washing stations present in the production area	P	A	P	P	P (insufficient number of hand washing stations)
Staff canteens not appropriately located; employees stored their food in the production areas	P	P	A	A	A
Non compliant personal protective equipment usage/condition	P (gloves not in clean condition; none of the employees wore hair nets)	P (not all employees were properly wearing gloves; none of the employees wore hair nets)	P (not all employees wore hair nets)	A	P (gloves not in clean condition; not all employees wore hairnets)

P: Presence of non-conformity.

A: Absence of non-conformity.

For utilities, only company E (20%) had established requirements for water and separate supply of potable water, while A, B, D (60%) used a reverse osmosis water purification device (Table 3). Company C (20%) did not take any additional measures to the non-potable water supply. None of the companies had established requirements for air used in direct contact with packaging material.

Regarding waste, none of the companies had clearly identified bins to differentiate between production and non-production waste. Similar findings regarding non-compliance was observed in catering services in northern Spain, where repeated deviations included absence of dustbins with pedal (40%) in the preparation and cooking areas [13]. In addition, incorrect handling of waste was found in all foodservices evaluated in Portugal [21].

Equipment hygienic design and food packaging contact surfaces were not suitable in all companies. Company B (20%) had the correct design however placed leather and scotch tape on the final counters. Company E (20%) had wooden surfaces covered with nylon and the remaining companies A, C, D (60%) did not place any measures for non-compliant surfaces (Table 3).

For maintenance, companies C, D, E (60%) had a system of planned preventive maintenance for the machines used whereas A and B (40%) only performed corrective maintenance on need basis (Table 3). Similarly, for catering services in northern Spain, one of the most important deviations were the lack of a maintenance plan for facilities and equipment (40%) [13].

Concerning measures for prevention of contamination, only company E (20%) carried out and documented a hazard analysis, since they are planning to get ISO 22000 certification. Proper documentation was missing in most companies, since none had food safety management system certification. On the other hand, in children's nurseries in Warsaw, results showed that the level of compliance with both good manufacturing practices (GMP)/good hygiene practices (GHP) and hazard analysis of critical control points (HACCP) standards was high in respect of documentation not practice [24]. For microbial contamination, companies A and C (40%) had no implemented measures (Clause 4.7, Table 2) (Table 3). Even though company E was the only company with documented hazard analysis including microbial hazards; it did not fully mitigate the risk along with companies B and D (60%). Few considerations were made in this regard such as changing compressed air filter in company, facility layout air flow from least contaminated to most contaminated and proper cleaning. Similar to our findings regarding contamination, repeated deviation included food products in contact with the floor (40%) for catering services in northern Spain [13].

In cleaning clauses, companies C and D (40%) had daily cleaning schedule after production and proper monitoring program (Company D). Companies A and B (40%) had none (Clause 4.8, Table 2, Table 3). Company E had a documented cleaning program but did not fully monitor its effectiveness. Similarly, in food businesses assessed in Turkey, failure in food-handling practices were related to the cleaning and sanitation of utensils, equipment, and cleaning and sanitizing of the food contact surface was not observed in 39 of the food businesses [19]. In addition, non-conformities were observed in all foodservice units in Portugal on cleaning and disinfection practices of equipment and facilities [21]. In all companies cleaning agents were clearly identified, stored separately and used only in accordance with the manufacturer's instructions.

For pest control, companies B, C and D (60%) outsourced their pest control programs, which showed good compliance (Clause 4.9, Table 2, Table 3). However, partial or non-compliance was observed for covering external doors, windows or ventilation openings to prevent entry of pests. Company B also did not have enough rodent bait stations and traps. The remaining companies A and E (40%) performed in-house control; which showed poor compliance (Clause 4.9, Table 2).

Regarding personnel hygiene and facilities, all personnel, visitors and contractors in companies B and E (40%), were required to comply with the visual instructions and signs (posters at the entrance door, entrance only allowed with presence of personnel in charge, hair nets present at the door). In company D (20%), oral instructions were given to visitors to wear hair nets. The remaining companies A and C (40%) did not have any procedure in place (Clause 4.10, Table 2, Table 3). Majority of the companies (A, C, and D) (60%) did not have any hand washing stations present in the production area. Company B (20%) had one hand washing station for a shift of minimum fifteen employees and hand washing was done in staff wash room. Company E (20%), had 2 hand washing stations but this was still not sufficient based on the size of the facility and number of employees (25 employees). While no specific number of basins is recommended, this number should take into account the number of handlers as they might be discouraged from washing hands if they have to wait or if the station is not located where needed [29]. Similar to our study, lack of specific sinks for hand washing (73.3%) was a repeated deviation in catering services in northern Spain [13], and in hospital food service in Turkey in addition to inadequate numbers of toilets for food service staff and overall mean score on personnel hygiene scores were 55.3% ± 14.7% [18]. In addition, lack of dedicated hand washing sink in food preparation and distribution areas with proper drying setting was observed in 60% of meal production units at schools [21].

Staff canteens were not appropriately located in companies A and B (40%), they were not isolated by closed doors (Company B) and their access route was through production area (Company A) (Clause 4.10, Table 2, Table 3). In addition, employees stored their food in the production areas in a refrigerator (company A) or under the production counter (company B). Companies D and E (40%) had dedicated space but employees’ behavior and practices were not fully compliant. Only company C (20%) had a more spacious facility with properly designed and situated canteen. Similarly, food businesses assessed in Turkey, employees were observed eating and drinking in the food-service areas [19].

Gloves were not in clean condition in companies A and E (40%) but were used properly in companies C and D (40%). Company B (20%) provided gloves that were in clean conditions for majority of employees; but some employees did not comply. Hair nets were worn by all employees of Company D (20%) whereas not all employees complied and wore them in companies C and E (40 %), and none in company A and B (40%). Personal cleanliness was most compliant in company D, since there is a designated employee for follow up, however full compliance was not granted due to the lack of hand washing stations in production areas. Additional preventive measures such as face masks were worn in companies C and E (40%) since visits were conducted during Corona virus disease of 2019 (COVID-19) pandemic, which increased overall PRP compliance (Table 3).

### 3.3 Assessment of hygienic status of contact surfaces

The production lines within these food packaging facilities lack full automation and involve numerous junctures necessitating human intervention and interaction with surfaces. Poor sanitation of food contact surfaces, equipment, and processing environments have been an important factor in foodborne outbreaks [30]. Assessing the hygienic status of contact surfaces is a common objective measurement of assessing cleaning practices in factories [13,[30], [31], [32]].

To assess the hygienic status of surfaces in contact with food packaging, 2 surface swab samples were collected from all the companies with a total of 10 samples, as shown in Table 4 and Table 8.

**Table 4:** Microbiological counts of swab samples collected from surfaces in contact with food packaging materials at the end of the production line (n = 10).

Company	Surface description	Packaging type	Total Viable Count (log CFU/cm^2^ ± SD^a^)	*Enterobacteriaceae* (log CFU/cm^2^±SD^a^)
**A**	Surface 1	Ketchup gallon (PP^c^)	1.3 ± 0.02	<DL^b^
	Surface 2	Lemon substitute bottle (HDPE^d^)	0.4 ± 0.01	<DL^b^
**B**	Surface 1	Smoked Salmon Vacuum Bag (PE^e^)	<DL^b^	<DL^b^
	Surface 2	Frozen Fish Bag (PE^e^)	1.3 ± 0.02	<DL^b^
**C**	Surface 1	Beef Vacuum Bag (PET^f^)	0.4 ± 0.01	<DL^b^
	Surface 2	Zip-lock Bag (LDPE^g^)	<DL^b^	<DL^b^
**D**	Surface 1	Cheese Container (PET^f^)	1.3 ± 0.02	<DL^b^
	Surface 2	Dessert Container (PET^f^)	1.1 ± 0.02	<DL^b^
**E**	Surface 1	Multipurpose vacuum bag (PET^f^)	<DL^b^	<DL^b^
	Surface 2	Multipurpose vacuum bag (PET^f^)	1.3 ± 0.02	<DL^b^

a Standard Deviation.

b Below Detection Limit: 10 CFU/25 cm^2^.

c PP: Polypropylene.

d HDPE: High-density polyethylene.

e PE: Polyethylene.

f PET: Polyethylene terephthalate.

g LDPE: Low-density polyethylene.

Surface cleanliness was evaluated based on acceptable limits of ≤0.6 log CFU/cm^2^ and 0 log CFU/cm^2^ for TVC and *Enterobacteriaceae,* respectively [13,32]. There are no unified standards and set limits for contact surfaces cleanliness and the ones used were based on established criteria in other studies [13,32]. Non-conforming results were observed in 50% (5/10) of the surfaces (Table 4). Highest counts of 1.3 log CFU/cm^2^ were from metal box for ketchup gallons made from polypropylene (PP) (Company A), scotch taped counter for Polyethylene (PE) (Company B), Cheese container machine line made from Polyethylene terephthalate (PET) (Company D), bag machine forming shoulder surface (Company E) (Table 4). Other non-conforming results at 1.1 log CFU/cm^2^ were observed on the dessert container made from PET machine line (company D) (Table 4). Lower conforming levels at 0.4 log CFU/cm^2^ were from metal box collector for lemon substitute bottles made from High-density polyethylene (HDPE) (Company A) and scotch taped counter for ready to eat beef vacuum bag (Company C) (Table 4). Remaining conforming results were below detectable limit for swabs from end of line leather counter (Company B), wooden surface (Company C), and roller of roll slitting machine (Company E) (Table 4). Regarding *Enterobacteriaceae*, all samples were acceptable and showed results below the detection limit (DL) (Table 4).

Company C had 100% (2/2) compliant results for surface samples. Conformance varied between below limit of detection for wooden surface and 0.4 log CFU/cm^2^ for scotch taped counter (Table 4). The scotch taped counter was made from one layer and had no to barely visible gaps between lines of tape; whereas the wooden surface was smooth and easily cleanable.

Companies A, B, and E had 50% (1/2) compliant results for surface samples. Samples taken from the same company A were non-conforming for the metal box for ketchup gallon and conforming for the collector of lemon substitute bottles (Table 4). In company B, compliance was observed in counter covered with leather and non-compliance in scotch taped counter for frozen fish bag (Table 4). Finally, in company E, compliance was observed in roller of roll slitting machine and non-compliance in bag machine forming surface (Table 4). Where variant results were found in the same companies; it was observed that the surfaces tested had different levels of smoothness and ease of cleaning i.e. ridges in the metal box for ketchup gallons made from PP collection (Company A), smooth surface provided by the leather cover versus non-uniform scotch tape showing gaps between layers (Company B), the frequency and ease of cleaning the slitting roll versus the difficulty in cleaning the forming shoulder (Company E). Along with adherence to proper hygienic design and use of suitable contact materials, there should be constant checking and replacement of damaged surfaces [13].

Whereas, company D, showed 100% (2/2) non-compliant results for surface samples(Table 4).

It was observed that equipment hygienic design and food packaging contact surfaces were not fully compliant in all companies where scores varied for equipment suitability, cleaning and maintenance between 27% (company A) and 58% (company C) (Clause 4.5, Table 2). In addition, proper cleaning programs should be put in place, resources (utensils and detergents) should be supplied, and proper monitoring and verification programs should be followed [13].

Following the same criteria, the percentage of conformity was lower for TVC and higher for *Enterobacteriaceae* in catering services located in northern Spain (n = 15). 86.7% and 96.7% of food contact surfaces were conforming to the mesophilic aerobes (MA) and *Enterobacteriaceae* criteria, respectively, showing good cleaning and disinfection standards [13]. Higher counts of TVC and *Enterobacteriaceae* were related to lower total checklist scores [13]. Our study results were also lower than a study conducted in Navarra (Spain) (n = 600) to assess surface hygiene control in two catering services where only 15.9% of the surfaces exceeded the limit of 0.6 log CFU/cm^2^ for TVC [32].

Following more lenient criteria (TVC <1.3 log CFU/cm^2^), higher percentage of conformity (74%) was observed on food contact surfaces (n = 4611) in hotels (n = 280) in Spain [31].

### 3.4 Assessment of hygienic status of employee hands

Assessing the hygienic status of employee is a common objective measurement of assessing personnel hygiene practices in factories [13,16,33,34]. Hand samples were taken from end of line employees in all companies with a total of 10 samples as shown in Table 5 and Table 8.

**Table 5:** Microbiological counts of swab samples collected from employee hands in contact with food packaging materials at the end of the production line (n = 10).

Company	Surface description	Packaging type/Food application	TVC (log CFU/hand ± SD^a^)	*Enterobacteriaceae* (log CFU/hand± SD^a^)
**A**	Operator1	PP^c^/Ketchup gallon	3.0 ± 0.04	<DL^b^
	Operator 2	HDPE^d^/Lemon substitute bottle	1.7 ± 0.03	<DL^b^
**B**	Operator 1	PE^e^/Smoked Salmon Vacuum Bag	2.9 ± 0.04	<DL^b^
	Operator 2	PE^e^/Frozen Fish Bag	3.3 ± 0.05	<DL^b^
**C**	Operator 1	PET^f^/Beef Vacuum Bag	4.3 ± 0.06	<DL^b^
	Operator 2	LDPE^g^/Zip-lock Bag	3.2 ± 0.05	<DL^b^
**D**	Operator 1	PET^f^/Cheese Container	2.6 ± 0.04	<DL^b^
	Operator 2	PET^f^/Dessert Container	4.4 ± 0.07	<DL^b^
**E**	Operator 1	PET^f^/multipurpose vacuum bag	1.3 ± 0.02	<DL^b^
	Operator 2	PET^f^/multipurpose vacuum bag	3.1 ± 0.05	<DL^b^

a Standard Deviation.

b Below Detection Limit: 10 CFU/hand.

c PP: Polypropylene.

d HDPE: High-density polyethylene.

e PE: Polyethylene.

f PET: Polyethylene terephthalate.

g LDPE: Low-density polyethylene.

There are no unified set limits for cleanliness of hand employees. Studies that aimed at assessing hand swabs to determine effectiveness of hand washing, only determined presence or absence of bacteria [13,16,35]. In other studies, the set limits for hand swabs were the same as limits used for contact surface conformity assessment [33,34]. The findings of our study were the same whether adopting the presence or absence criteria or the less stringent one with acceptable limits of ≤0.6 and 0 log CFU/hands for TVC and *Enterobacteriaceae,* respectively [13,32].

None of the hand swab samples was conforming for TVC that was present in all samples above the acceptable limit but all the samples were below the detectable limit for *Enterobacteriaceae* (Table 5).

All the companies had generally low scores ranging from 7 to 57% for personnel hygiene and facilities (clause 4.10, Table 2). This was mainly attributed to the lack of or insufficient number of handwashing stations. The unsatisfactory results emphasize the need for adequate supply of hand washing stations, regular training for employees on proper hand washing technique and provision of soap, disposable tissues, and sanitizer. This is supported by previous work in Spain that showed that mesophilic aerobic microorganisms, *Staphylococcus* spp. And *Enterobacteriaceae* were respectively present in 91.3, 53.3 and 22.8%, of the food workers’ hands before washing, while significant reduction of microbial load was obtained on all bare or gloved hands after washing [13].

In addition to hand washing, training should include all general hygiene practices e.g. refraining from touching their face, food, drinks, waste bins, and cigarettes, proper usage of gloves, proper nail trimming, and frequency of hand washing. The proper adherence to these practices should also be monitored regularly by designated employee to ensure training efficacy. It is important for employees to understand the sources of contamination and the route of spread from their hands to end product [9,13,16]; especially since all packaging material are counted and sorted manually after processing. Similarly, *Enterobacteriaceae, Escherichia coli, and Staphylococcus aureus* were present in 62.1, 7.5 and 26.6% of food handlers' samples during food preparation for collective meals in Spain, respectively [16]. In Malaysia, the aerobic plate count reported on the employee's hands was only 20, 28, and 29% acceptable before, after, and during ready to eat preparation, respectively [33]. In addition, it must be pointed out that TVC counts varied between one employee and another in the same company. For example, results varied between 2.6 and 4.4 log CFU/hands in company D and between 1.3 and 3.1 log CFU/hands in company E (Table 5). Those differences showed the importance of strengthening food safety culture, employee's behavior and refresher trainings; especially that only 3 companies (60%) reported conducting more than 1 food safety training per year. The significance of effectiveness of PRPs implementation can show tangible results on the reduction of bacterial load. This was demonstrated in a study conducted in Serbia in meat processing plants and meat retail, where hand swab acceptability (TVC ≤2.7 log CFU/cm^2^), increased from 47.29 to 48.9 to 97–98.96% before and after HACCP implementation, respectively [34].

### 3.5 Assessment of hygienic status of food packaging samples

Packaging samples used for RTE products were collected from the 5 companies (a total of 10 samples) to check their hygienic quality and from retail market (a total of 20 samples) to simulate different routes of contamination along the supply chain.

Limited information is available about the microbiological criteria for packaging material. The Food and Drug Administration (FDA) developed guidelines for single-service containers and closures, used for packaging pasteurized milk and/or milk products dairy products [36], but there are no specific standards or recommendations for other types of packaging.

The microorganisms should be absent or as low as possible since their presence can be a concern when the packaging will get in contact with the food.

For the samples collected during on-site inspections, TVC and yeasts and molds were detected in 20% (2/10) of the samples (Table 6). *Enterobacteriaceae* were not detected in all samples (Table 6). In company D, TVC (0.7–0.8 CFU), yeast and molds (0.3–0.8 CFU) were detected in both dessert and cheese PET containers (Table 6, Table 8). In addition, the score of company D was only 41% on the clause related to prevention of contamination (Clause 4.7, Table 2).

**Table 6:** Microbiological counts of food packaging samples collected during on-site inspection (n = 10).

Company	Packaging Sample/Food application	Test method	Total Viable Count (log CFU/Container or/cm^2^ ± SD^a^)	*Enterobacteriaceae* (log CFU/Container or/cm^2^ ± SD^a^)	Yeasts & Molds (log CFU/Container or/cm^2^ ± SD^a^)
**A**	PP^d^/Ketchup Gallon	Rinse test	<DL^b^	<DL^b^	<DL^b^
	HDPE^e^/Lemon Substitute Bottle	Rinse test	<DL^b^	<DL^b^	<DL^b^
**B**	PE^f^/Smoked Salmon Vacuum Bag	Swab test	< DL^c^	< DL^c^	<DL^c^
	PE^f^/Frozen Fish Bag	Swab test	< DL^c^	< DL^c^	< DL^c^
**C**	PET^g^/Beef RTE Vacuum Bag	Swab test	< DL^c^	< DL^c^	< DL^c^
	LDPE^h^/Dairy RTE Vacuum Bag	Swab test	< DL^c^	< DL^c^	< DL^c^
**D**	PET^g^/Cheese Container	Rinse test	0.7 ± 0.01	<DL^b^	0.3 ± 0.0
	PET^g^/Dessert Container	Rinse test	0.8 ± 0.01	<DL^b^	0.8 ± 0.01
**E**	PET^g^/Ready To Eat Humus bag	Swab test	< DL^c^	< DL^c^	< DL^c^
	PET^g^/Multipurpose Vacuum bag	Swab test	< DL^c^	< DL^c^	< DL^c^

a Standard Deviation.

b Below Detection Limit: 1 CFU/Container (Rinse test).

c Below Detection Limit: 10 CFU/25 cm^2^ (Swab test).

d PP: Polypropylene.

e HDPE: High-density polyethylene.

f PE: Polyethylene.

g PET: Polyethylene terephthalate.

h LDPE: Low-density polyethylene.

As for the samples collected from the retail market, TVC were detected in 95% (19/20) of samples (Table 7). The highest TVC count was 1.6 log CFU/Container for one container from salad/bakery/meat category (Table 7). Yeasts and molds were detected in 65% (13/20) of the samples with the highest value in a PP dairy/ice cream sample at 1.1 log CFU/Container (Table 7) *Enterobacteriaceae* were below detection limit in all the samples (Table 7).

**Table 7:** Microbiological counts of food packaging samples collected from retail market using rinse test method (n = 20).

Category	Total Viable Count log CFU/Container	*Enterobacteriaceae* log CFU/Container	Yeasts & Molds log CFU/Container
1. Dairy/Ice Cream Containers (PP^b^)	1.4	< DL^a^	1.1
	0.3	< DL^a^	< DL^a^
	0.7	< DL^a^	0.3
	1.0	< DL^a^	0
2. Salad/Bakery/Meat Containers (PET^c^)	1.4	< DL^a^	0
	0.3	< DL^a^	< DL^a^
	1.6	< DL^a^	0.3
	0.7	< DL^a^	0
3. Mezze Containers (PET^c^)	1.3	< DL^a^	0.7
	4. 0.6	< DL^a^	0.3
	1.2	< DL^a^	0
	0	< DL^a^	0.3
5. Sauce Cups With Lids (PP^b^)	0.9	< DL^a^	0.3
	6. 0.6	< DL^a^	0.3
	< DL^a^	< DL^a^	< DL^a^
	0.3	< DL^a^	< DL^a^
7. Dessert/Fruit Cups With Lid Opening (PET^c^)	0.3	< DL^a^	< DL^a^
	1.3	< DL^a^	< DL^a^
	0.3	< DL^a^	< DL^a^
	1.4	< DL^a^	0.3

a Below Detection Limit: 1 CFU/Container (Rinse test).

b PP: Polypropylene.

c PET: Polyethylene terephthalate.

**Table 8:** Percentage of acceptable and unacceptable levels of TVC, Enterobacteriaceae, Yeast and Molds in contact surfaces and employee hands during inspection visits.

			Company				
Variable	Parameter/Acceptable Limit	Criterion	A	B	C	D	E
Hygienic Status of contact surfaces	TVC/≤ 0.6 log CFU/cm^2^ [13,32]	% of acceptable levels (n/N^a^)	50(1/2)	50(1/2)	100(2/2)	0(0/2)	50(1/2)
		% of unacceptable levels (n/N^b^)	50(1/2)	50(1/2)	0(0/2)	100(2/2)	50(1/2)
	*Enterobacteriaceae*/Absent [13,32]	% of acceptable levels (n/N^a^)	100(2/2)	100(2/2)	100(2/2)	100(2/2)	100(2/2)
		% of unacceptable levels (n/N^b^)	0(0/2)	0(0/2)	0(0/2)	0(0/2)	0(0/2)
Hygienic status of employee hands [13,32]	TVC/≤ 0.6 log CFU/cm^2^ [13,32]	% of acceptable levels (n/N^a^)	0(0/2)	0(0/2)	0(0/2)	0(0/2)	0(0/2)
		% of unacceptable levels (n/N^b^)	100(2/2)	100(2/2)	100(2/2)	100(2/2)	100(2/2)
	*Enterobacteriaceae*/Absent [13,32]	% of acceptable levels (n/N^a^)	100(2/2)	100(2/2)	100(2/2)	100(2/2)	100(2/2)
		% of unacceptable levels (n/N^b^)	0(0/2)	0(0/2)	0(0/2)	0(0/2)	0(0/2)

a n/N: number of acceptable samples/total number of samples of each category.

b n/N: number of unacceptable samples/total number of samples of each category.

Overall counts of samples taken from the retail market were higher than the ones from companies for TVC and yeasts and molds; however, *Enterobacteriaceae* results were all the same. This can be attributed to the additional sources of contamination during transportation, distribution, handling, storage, and retail display and highlight the importance of control measures along all the supply chain.

The counts of TVC and yeasts and molds that were detected; were present on packaging material intended to be in direct contact with RTE food (cheese, dessert, salad, bakery, meat) without any further sterilization process. The effect of these counts, their interaction with stored foods, presence of nutrients and other growth factors can increase the bacterial load in the food [7,37]. Packaging materials can thus be a source of microflora which can influence food products in terms of safety and quality [37]. A study in Egypt demonstrated the effect of packaging container (plastic and cardboard) contamination on the bacteriological profile of Egyptian soft cheese [7]. Plastic containers and cardboard laminated packages had mean Aerobic plate counts of 4.49 × 10^3^ CFU/Package (3.6 log CFU/package) and 1.17 × 10^3^ CFU/cm^2^ (3.1 log CFU/cm^2^), respectively [7]. Results showed a significant positive relationship between the level of soft cheese contamination (especially with aerobic plate count, aerobic spore-formers and *Staphylococci*) as a result of their packaging in plastic containers and cardboard laminated containers [7]. The increase was explained by the possibility of a certain permeability of the packaging used [7].

Also another study explored the microbial transfer dynamics from packaging to packed peaches in relation to the packaging used [8]. The quality of 30 peaches was evaluated, after sanitizing and before packaging [8]. All levels of *Pseudomonas* spp., yeasts and *E. coli* were under the detection limits. The data showed a higher contamination frequency of the fruits packed in plastic than in cardboard [8]. By increasing the storage temperature and the number of lesions in peaches, the probability of transferring of *Escherichia coli* from packaging materials to fruits increased [8]. For *Pseudomonas*, the contamination levels detected on fruits packaged in plastic were significantly higher compared to those found on fruits packed in cardboard, independently of the considered variables [8].

In addition, several other studies showed higher microbial counts on food packaging materials. In Bangladesh flexible intermediate bulk food containers showed counts of TVC (2.06 ± 0.08 log CFU/unit) and *Staphylococcus* spp. (1.78 ± 0.01 log CFU/unit) in the spout top of the bags; indicating handling by employees with contaminated hands [17]. On the other hand, the spout bottom was found clean but after enrichment, the presence of stressed coliforms was evident [17]. However, *Salmonella* spp. Was not found in any samples analyzed [17]. In a production line for plastic caps for soft drink bottles filled according to the aseptic method, the raw materials including the base resin, the master batch, and the linear material indicated an average of 0.9 filamentous fungi per sample [38]. In another study, eight food grade cling film samples purchased from supermarkets in Iran (n = 15), had bacterial contamination with three different bacterial strains of *Bacillus* spp. (except *B. anthracis*), *Klebsiella* spp. And coagulase positive *Staphylococcus* spp [7]. Therefore, it is very important to monitor, test and control the hygienic status of packaging materials to ensure the safety and quality of food products. In addition, the use of new innovative technologies such as active antimicrobial packaging can be a promising approach to protect both the food and the packaging material itself [[39], [40], [41], [42]].

## 4 Conclusions

PRPs assessment and verification activities showed the need for companies to strengthen their PRP planning and implementation such as: proper cleaning programs, frequent training on personnel hygiene and hand washing practices. Recommendations extend to all companies visited since even the companies with the highest scores had contamination problems. Food packaging companies should direct more effort towards monitoring, enhancing PRPs and allocating budget for these programs to implement food safety management programs not only quality management programs. Effectiveness of implementation of PRPs should be assessed by verification activities on a planned routine basis. Therefore, verification activities should not be limited to one-time testing, since end product testing in general has several limitations. Food packaging companies should take into consideration within their scope the effect of distribution and various points in the supply chain especially when their product is being sold at retail markets. Hygienic status should be maintained where proper wrapping and sealing of products should be carried out to prevent contamination along the route before it reaches the consumer. Food packaging hygienic status has a great impact on the quality and safety of food especially when used without further sterilization for temperature sensitive food products. Our study was conducted on a small sample of factories due to confidentiality concerns; but it added to the body of science shedding light on the poor status of PRPs and the need to collect more data and assess compliance in both packaging and food companies. Studies should also assess status of exporters supplying food packaging worldwide. More studies should evaluate other testing parameters such as the presence of spores that withstand heat exerted during processing and develop antimicrobial packaging to control the presence of microorganisms on the packaging materials. Finally, clearer legislations and guidelines should be issued by national and international bodies for the variety of packaging material used; since guidelines are mainly present for chemical parameters such as chemical migration. Guidelines should include specific bacterial parameters to be tested, testing method, cut-off points for acceptability (risk-based and related to the type of packaged food), and implementation of best practices for food packaging companies.

## Funding

This work was supported by 10.13039/100012984Notre Dame University-Louaize, the Industrial Research Institute (IRI) and the Euro-Lebanese Centre for Industrial Modernization (ELCIM) at the 10.13039/501100004113Lebanese University Campus Hadeth (Baabda). The open access funding was provided by the Qatar National Library.

## Ethics statement

The study complies with all regulations and informed consent was obtained from the participants in collecting the samples.

## Author contribution statement

Marilyn Abdessater: Performed the experiments; Analyzed and interpreted the data; Wrote the paper. Fady Fayyad, Joseph Matta: Contributed reagents, materials, analysis tools or data. Layal Karam: Conceived and designed the experiments; Analyzed and interpreted the data; Wrote the paper.

## Data availability statement

The data that has been used is confidential.

## Declaration of competing interest

The authors declare that they have no known competing financial interests or personal relationships that could have appeared to influence the work reported in this paper.
